# From Cellular to Metabolic: Advances in Imaging of Inherited Retinal Diseases

**DOI:** 10.3390/diagnostics15010028

**Published:** 2024-12-26

**Authors:** Deepika C. Parameswarappa, Ashwini Kulkarni, Niroj Kumar Sahoo, Srikanta Kumar Padhy, Sumit Randhir Singh, Elise Héon, Jay Chhablani

**Affiliations:** 1 Ophthalmology and Vision Sciences, Hospital for Sick Children, University of Toronto, Toronto, ON M5S 1E8, Canada; 2 Anant Bajaj Retina Institute, LV Prasad Eye Institute, Vijayawada 521134, India; 3 Anant Bajaj Retina Institute, LV Prasad Eye Institute, Bhubaneswar 751024, India; 4 Akhand Jyoti Eye Hospital, Bihar 841219, India; 5 Genetics and Genome Biology, SickKids Research Institute, Toronto, ON M5G 1E8, Canada; 6 UPMC Eye Centre and Choroidal Analysis and Research (CAR) Lab, University of Pittsburgh, Pittsburgh, PA 15213, USA

**Keywords:** inherited retinal diseases, cellular imaging, adaptive optics, fluorescence lifetime imaging ophthalmoscopy, PS-OCT, metabolic imaging, retina, imaging

## Abstract

**Background**: Inherited retinal diseases (IRDs) are a genetically complex group of disorders, usually resulting in progressive vision loss due to retinal degeneration. Traditional imaging methods help in structural assessments, but limitations exist in early functional cellular-level detection that are crucial for guiding new therapies. **Methods**: This review includes a systematic search of PubMed and Google Scholar for studies on advanced imaging techniques for IRDs. **Results**: Key modalities covered are adaptive optics, fluorescence lifetime imaging ophthalmoscopy, polarization-sensitive optical coherence tomography, optoretinography, mitochondrial imaging, flavoprotein fluorescence imaging, and retinal oximetry. Each imaging method covers its principles, acquisition techniques, data from healthy eyes, applications in IRDs with specific examples, and current challenges and future directions. **Conclusions**: Emerging technologies, including adaptive optics and metabolic imaging, offer promising potential for cellular-level imaging and functional correlation in IRDs, allowing for earlier intervention and improved therapeutic targeting. Their integration into clinical practice may significantly improve IRD management and patient outcomes.

## 1. Introduction

Inherited retinal diseases (IRDs) are a group of clinically and genetically heterogeneous disorders. IRDs predominantly affect the outer retinal layers, i.e., photoreceptors, and retinal pigment epithelium with or without affection of inner retinal layers and choroid [1,2,3]. More than 300 genes are known to cause IRDs (https://retnet.org) and lead to legal blindness in the majority of the affected patients. IRDs are broadly categorized as progressive vs. stationary depending on the disease course. Based on the topography of retinal involvement, IRDs can be central or generalized [4]. Retinal imaging in IRDs is crucial as it helps in the characterization of disease structure (phenotype) and in assessing the progression. Most commonly performed conventional retinal imaging in IRDs include fundus photography, optical coherence tomography (OCT), and fundus autofluorescence (FAF). The function of the retina in IRDs is assessed by electrophysiological tests, visual fields, microperimetry, and full-field stimulus tests.

Gradually progressive IRDs need a definitive assessment at the cellular level before structural changes arise in conventional imaging or to detect changes before a particular cell type is dead. This will help in early detection, better prognostication, and possible therapies. The current retinal imaging modalities used in clinical practice face several challenges, including the inability to detect changes before functional loss occurs, the presence of overlapping or similar features in advanced stages of IRDs, a lack of clear correlation between structure and function, and the inability to image the retina at the cellular level. In recent years, advanced imaging modalities providing information at the cellular level are available, like adaptive optics (AO). However, the use of AO as a clinic-based test is still challenging. Functional correlation to AO findings is emerging. The conventional functional test, electroretinogram (ERG), offers an overview of global retinal function but often yields undetectable responses in the majority of the IRD phenotypes, despite the presence of residual functional cones or rods. This limitation makes it challenging to monitor disease progression once ERG signals become undetectable. In contrast, the newer advanced imaging modalities discussed here enable detailed structure/functional analysis of photoreceptor cells, within localized areas of preserved retinal function.

The retina is a highly metabolic structure with photoreceptors using the maximum oxygen among all cells in the body. Hence, assessing the retinal metabolic demand in specific IRDs and detecting metabolic changes before the cellular changes is an ideal approach toward these disorders. US Food and Drug Administration approved the first gene replacement therapy (Voretigene neparvovec-rzyl; Luxturna™ Spark^®^ Therapeutics, Inc. Philadelphia, PA, USA) for biallelic RPE65-related retinal dystrophy in 2017 [5]. Luxturna has shown promising results with functional improvement in *RPE65*-related Leber congenital amaurosis (LCA) and early onset retinal dystrophy (EOSRD). Approval of Luxturna has opened the door for many therapeutical approaches for IRDs, which were previously thought to be untreatable. Other newer therapies under various phases of clinical trial include gene-related therapies for >20 monogenic IRDs, cell-based therapies, optogenetics, and oral pharmacological products [1,6,7]. Therapies are targeted at either the cellular level or at the level of visual cycle products. This has mandated accurate structural and functional analysis at the cellular level as an outcome measure.

The current review focuses on newer imaging modalities that can image the retina at the cellular level in IRDs and correlate to functional changes. The review also covers the latest metabolic imaging mostly used as an investigational modality.

## 2. Methodology

The literature review included a systematic review of PubMed and Google Scholar databases. The search terms used were “imaging advances in inherited retinal diseases”, “fluorescence lifetime imaging ophthalmoscopy (FLIO)”, “updates in adaptative optics (AO)”, “near infra-red reflectance imaging”, “near infra-red fundus autofluorescence”, “polarization sensitive OCT (PS-OCT)”, “polarization diversity OCT(PD-OCT)”, “optoretinography”, “mitochondrial imaging”, “flavoprotein fluorescence (FPF) imaging”, “retinal oximetry”, “detection of apoptosing retinal cells (DARC)”, and “advances in imaging of most common IRDs” (like retinitis pigmentosa, Stargardt diseases, cone dystrophy, etc.). The search data were initially customized to focus on the recent updates in the past two decades (from year January 2000 to June 2024). The original articles with proof-of-concept studies before the year 2000 were cross-referenced accordingly. Review articles and retrospective and prospective studies in the English language were extracted (179 in number) with a review of abstracts and citations. Each imaging will be detailed in Section 2.1, Section 2.2, Section 2.3, Section 2.4, Section 2.5, Section 2.6, Section 2.7, Section 2.8 and Section 2.9 with its principle, technique/image acquisition, data in healthy eyes, role in IRDs with disease-specific examples, and current challenges with future directions.

### 2.1. Fluorescence Lifetime Imaging Ophthalmoscopy (FLIO)

Fluorescence lifetime imaging ophthalmoscopy is a fundus autofluorescence (FAF)-based non-invasive retinal imaging. It works on the principle that all the fluorophores present inside the eye have specific excitation and emission spectrums that dictate their fluorescence lifetime [8,9,10]. The fluorescence lifetime, also referred to as FAF lifetime or decay time is defined as the time taken for detection of fluorescence signal post excitation by a particular wavelength. FLIO was originally based on fluorescence lifetime imaging microscopy (FLIM) employed for both systemic neoplastic and non-neoplastic disorders, which detects the metabolic changes at the cellular level such as oxidation, polarity, pH levels, and ion concentration, which help in understanding the micro-environment and biochemical functions [8,11,12]. The utility of FLIO has been demonstrated in various retinal disorders, including macular telangiectasia, macular hole, geographic atrophy, drusen, reticular pseudodrusen, diabetic retinopathy, and central serous chorioretinopathy [10].

***Principle of FLIO:*** There are various endogenous fluorophores in the retina and choroid with a broad range of absorption rate at 250 nm to emission rate at 700 nm. Each fluorophore molecule has a specific fluorescence lifetime that is independent of its concentration and majorly depends on the interaction with the surrounding microenvironment [8,9,10]. The major fluorophores present include lipofuscin, bis retinoids such as iso-A2E (bis-retinoid N-retinylidene-N-retinylethanolamine), iso-A2PE, the cis-trans isomers of A2E and A2PE, yellow carotenoids (zeaxanthin, mesozeaxanthin, lutein), melanin, collagen, elastin, lipid peroxidation end products, advanced glycation end products, amino acids, flavin, retinal, and nicotinamide adenine dinucleotide and its derivatives [10,13,14,15,16,17].

On excitation with a particular wavelength, the photons inside the fluorophores reach an excited state from their ground state. The average time, when the fluorophore molecule remains in the excited state before returning to the ground state is referred to as fluorescence lifetime. Lipofuscin is a widely studied fluorophore present in photoreceptor outer segments and retinal pigment epithelial cells [18,19]. It has an excitation spectrum of 440–470 nm with an emission spectrum of 510–700 nm [9,10,19,20]. The fluorescence lifetime indicates the metabolic state of the retina and depends on the molecule, local environment, and adjacent fluorophores. Currently, the fluorescence lifetime is composed of lifetimes from various fluorophores at different sites and does not measure layer-specific fluorophores [9,10,11].

***Technique and image acquisition*:** Heidelberg Retina Angiography (Heidelberg Engineering, HRA 2, Heidelberg, Germany), with confocal laser scanning ophthalmoscope (CSLO), has the setup for FLIO. A sensitive detector and a picosecond (ps) pulsed laser source with illumination excitation at 470 nm blue laser diode constitute the FLIO system [10,11]. Every single emitted fluorophore is detected by connecting the single photon counting detectors with single photon counting cards. There are two spectral sensitive channels for detection of emitted photons from the fluorophores: (1) Short spectral channel (SSC) with a wavelength of 498–560 nm, and (2) Long spectral channel (LSC) with a wavelength of 560–720 nm. The system acquires multiple repetitive scans at each pixel of the scan, 12 ns apart and the detected photons are plotted in a histogram. The histogram of florescence time is then adjusted with an exponential function. The shape of exponential decay time is influenced by movement artifacts if present, interaction between different fluorophores, and time distribution of the excited photons. The system also consists of an eye tracking system monitored by an infrared camera and corrects for eye movements. It captures about 9 × 9 mm of human retina covering a 30° field in the posterior pole with the fovea being the center. The central fixation can be altered to focus on the area of interest [21,22,23].

FLIO should be performed before the application of fluorescent dye, like for applanation tonometry, fluorescein, or indocyanine green angiography. A maximally dilated pupil is recommended with images captured in a completely darkened room to improve image quality with reduced light scattering to avoid any artifacts [24].

***FLIO in healthy eyes:*** FLIO in normal healthy eyes has a specific pattern in SSC and LSC (Table 1). With increasing age, there will be increased accumulation of fluorophores with longer decay times due to more lipofuscin and other visual cycle by-products. The mean fluorescence time increases by around 30 ps per decade (in phakic eyes) in both SSC and LSC [23,25,26]. Like age, features of the lens affect the fluorescence lifetimes. Significant lens opacities cause prolongation in fluorescence lifetimes and affect the quality of the FLIO image by light scattering [27].

***Role of FLIO in IRDs:*** The role of FLIO in inherited retinal diseases is emerging but still being investigated. The disease process of IRDs mostly affects the photoreceptors, and RPE with or without the involvement of choroids, which are the structures containing various fluorophores [10,28]. FLIO has been studied previously in Stargardt disease, retinitis pigmentosa, and choroideremia (Table 2).

**Figure 1:**
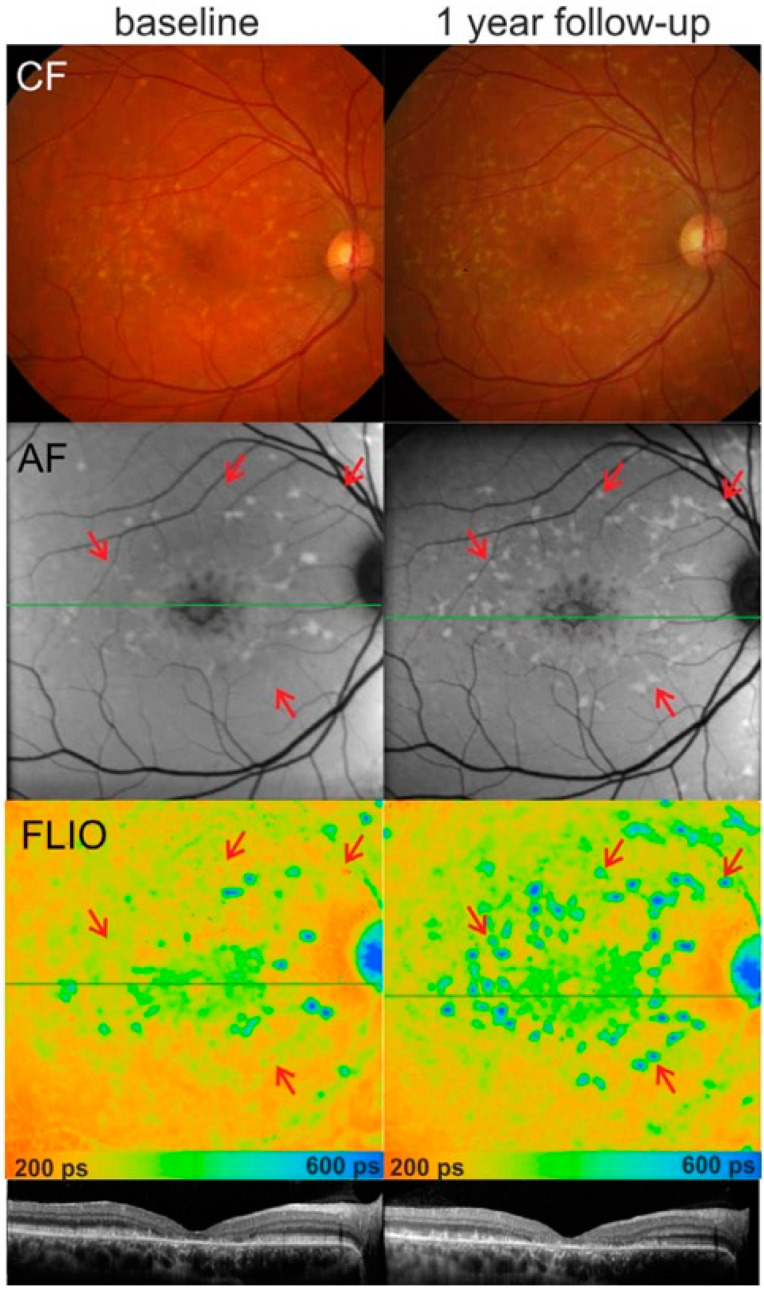
Disease progression within 1 year. Color fundus (CF), fundus AF, and FLIO (long spectral channel) with indicated lines of the optical coherence tomography scan (OCT) (baseline, **left**). A 1-year follow-up examination (**right**) shows clear disease progression with accumulation of hyperfluorescent flecks. The red arrows mark areas with short fluorescence lifetimes that converted to long lifetimes within 1 year. {This work is licensed under a Creative Commons Attribution-Noncommercial-NoDerivatives 4.0 International License. Reprinted with permission from iovs.arvojournals.org [29] (Dysli, C., S. Wolf, K. Hatz, and M.S. Zinkernagel, Fluorescence lifetime imaging in Stargardt disease: potential marker for disease progression. *Investigative Ophthalmology & Visual Science*, **2016,** *57*, 832–841)}.

*Stargardt disease (Figure 1)* with the presence of flecks and macular atrophy is the most common macular dystrophy phenotype due to pathogenic variants in the *ABCA4* gene. This results in abnormal accumulation of the A2E by-product, toxic to the RPE. On the evaluation of the flecks by FLIO vs. FAF intensity images, it was noted that changes in FLIO appear earlier than visible changes in FAF. The short fluorescence lifetime (242–322 ps) of early flecks was better identified in LSC. Patient’s symptoms were correlated with the onset of flecks with longer fluorescence lifetimes when the accumulation of bis retinoid was less. Flecks with short fluorescence lifetimes have more accumulation of the bis-retinoid by-products without the degeneration of outer retinal structures [29,30]. On the other hand, during the subsequent stage with long fluorescence lifetimes, there is more accumulation of lipofuscin, and degeneration of outer retinal structures sets in with the patient becoming symptomatic. It appears that flecks have longer lifetimes in the center of a fleck than the edges of the fleck [31].

Advanced Stargardt disease with atrophic areas of the retina has shown longer fluorescence lifetimes. A study has also shown changes in the fluorescence lifetimes of the flecks over the years with shorter to longer fluorescence lifetimes on disease progression. Solberg et al. compared the lifetimes of RPE atrophic regions in Stargardt vs. age-related macular degeneration patients (AMD). The Stargardt eyes showed a mean lifetime of 363 ± 26 ps in SSC and 393 ± 23 ps in LSC. However, these lifetimes in Stargardt eyes were significantly shorter than AMD eyes indicating disease-specific affection of the lifetimes with contribution from the aging process in AMD as well [32,33]. It was also noted that Stargardt patients showed similar lifetime values in the entire atrophic lesion indicating uniform fluorophore distribution [32]. FLIO can be a good tool to identify the onset of flecks before the onset of flecks in FAF and before the onset of patient symptoms.

*Retinitis pigmentosa (RP)* is a genetically heterogeneous rod–cone dystrophy with various clinical phenotypes and genetic subtypes. Studies comparing the features of the hyperautofluorescent ring in FAF to FLIO in RP eyes showed that areas outside of the ring had longer fluorescence lifetimes while areas inside the ring had shorter fluorescence lifetimes [34]. The central macular area with a shorter fluorescence lifetime had better visual acuity. It will be interesting to understand the changes in fluorescence lifetimes over the years and at the edge of the hyperautofluorescent ring. FLIO could become a biomarker of the functional retina for therapeutic interventions. FLIO in RP has also shown that retinal areas with loss of photoreceptors alone had slight prolongation of lifetimes, whereas the areas with loss of both the RPE and photoreceptors showed even more prolongation in lifetimes [34,35].

*Choroideremia* is an X-linked recessive disorder caused by a defect in the *CHM* gene. This disruption results in extensive chorioretinal degeneration with major involvement of choroid, RPE, and eventually the outer retinal structures. Conventional FAF depicts the areas with preserved RPE as hyperautofluorescent and areas with loss of RPE as hypoautofluorescent. However, the FAF does not guide in understanding the relative correlation between the loss of photoreceptors to RPE loss. Dysli et al. used FLIO in patients of choroideremia and showed the central macula with preserved retinal layers had the shortest fluorescence lifetimes. The areas with advanced RPE and photoreceptor loss showed prolonged fluorescence lifetime. The areas with RPE loss but preserved photoreceptors showed shorter fluorescence lifetime [36]. However, another study showed that there is a mixture of long and short fluorescence lifetimes in atrophic regions of choroideremia. This heterogeneous distribution was attributed to the presence of outer retinal tubulations, which were responsible for shorter fluorescence lifetimes in a few regions of the retina [37]. On progression of the disease, the conversion of the short fluorescence lifetimes to longer fluorescence lifetimes reflected the severity of the disease process over time [37,38].

***Challenges and Future directions:*** The strength of FLIO is in the detection of retinal changes before they are evident in FAF or even before the patient is symptomatic. FLIO can also provide the status of the visual cycle by-products and their correlation to various stages of the disease as an adjunct to the current FAF. The current challenges for FLIO include difficult data acquisition, data analysis, absence of standard algorithms, lack of data from healthy eyes, time-consuming, negative impact of media opacities like cataracts, and limited availability of the devices [9,10,27]. Image acquisition by FLIO in IRDs is further challenged when nystagmus is present. IRDs like Bestrophinopathies, other vitelliform disorders and pattern dystrophies, etc., need to be explored with FLIO. IRDs with a specific genotype are known to have a specific FAF pattern and this needs to be explored by FLIO. The genetic heterogeneity of IRDs poses extra challenges in assessing gene-specific patterns. The future direction in the FLIO imaging technique will likely be toward automated algorithms, easier data analysis, and understanding the fluorescence lifetimes of individual fluorophore layers by incorporating a wide range of spectral wavelengths in the device.

### 2.2. Adaptive Optics (AO)

AO is an in vivo ultra-high-resolution retinal imaging tool that captures the details of photoreceptors (PRs), RPE, and blood vessels at the cellular level. AO has shown important results in the imaging of IRDs with regard to natural history, quantification of photoreceptors, and its role in assessing the efficacy of therapies in clinical trials [39,40,41,42,43]. Conventional retinal imaging (OCT) has a transverse resolution of up to 20–30 µm size and an axial resolution of 5 µm size, whereas PRs are 2–5 µm in diameters that can be imaged with AO, which resolves at 1–2 µm size [39,44]. AO’s ability to detect microstructural changes before the appearance of functional changes has gained importance.

***Principle and mechanism*:** AO neutralizes the aberrations from various parts of the eye, thus increasing the resolution of the image at the cellular level. There are three components in the AO system: (1) Wavefront sensor to detect all the ocular aberrations. (2) Wavefront corrector to address the detected aberrations. (3) Control system that coordinates both sensor and corrector [43,45]. There are two AO systems: (a) Sensor-based optically complex and costly system that uses a deformable mirror. (b) Sensorless computational system that tackles the aberration by algorithms [42,46,47]. Though the speed of the sensor-based system is higher and has better resolution than the sensorless system, the image quality is noted to be comparable in both [48]. On the other hand, sensorless systems provide easy image acquisition.

**Figure 2:**
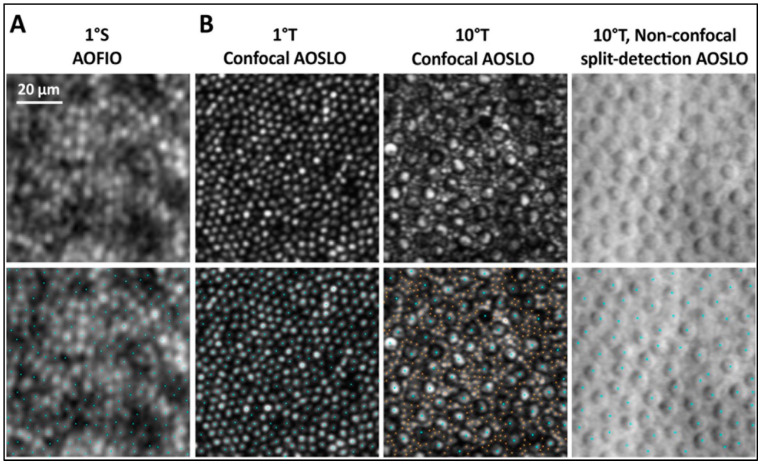
(**A**) AOFIO image of the normal cone mosaic at 1° superior to fixation. (**B**) AOSLO images of the photoreceptor mosaic in the parafovea using confocal imaging at 1° and 10° temporal to fixation using confocal and non-confocal split-detection. Individual cone and rod photoreceptors can be identified in the images (blue dots: cones, orange dots: rods). Reprinted with permission from [57] © Optica Publishing Group (Morgan, J.I., T.Y. Chui, and K. Grieve, Twenty-five years of clinical applications using adaptive optics ophthalmoscopy. *Biomedical Optics Express*, **2022**, *14*, 387–428.).

***Techniques, image acquisition, and analysis:*** Three widely used devices that can capture the retinal details include AO-scanning laser ophthalmoscopy(AOSLO), AO flood illuminated ophthalmoscopy (AOFIO), and AO-OCT [39,49]. AOSLO uses multiple raster scans with a focused single beam to assess the cone mosaic (at a small field of 0.2 to 1.0°, resolution of 100 um (axial) and 2.5 um (transverse)) [45,46]. A confocal aperture helps in better contrast and resolution. AOSLO is not commercially available [44,50]. AOFIO is the commercially available rtx1™ device (Imagine Eyes; Orsay, Paris, France). It captures a field of 4 × 4° (1.2 × 1.2 mm^2^) [51,52]. The images are captured by illuminating the area of interest with backscattered light; ~40 images are acquired in 2–4 s and montaged to produce a cone mosaic. Assessing photoreceptor cells beyond 6° by rtx1™ is challenging due to the increasing density of rods, and the central 2° fovea cannot be evaluated because of the high cone density [44]. AOFIO has less axial resolution due to a lesser capture time. AO-OCT is useful by increasing both axial and lateral resolution, thus giving a 3D retinal visualization and region-specific correlation [49,53]. Cone optoretinogram with phase-sensitive AO-OCT can detect functional changes even before visible affection in AO [54,55]. Other newer techniques in AO include the split detector technique, which can visualize the inner and outer segments of photoreceptors separately (Figure 2) [56,57]. The RPE mosaic can be visualized by AO dark mode, transscleral imaging, or in conjugation with AF (near-infrared and short wavelength) and ICG dye [58,59,60,61].

Transscleral optical phase imaging (TOPI) is a novel technique that utilizes the principle of oblique illumination through the sclera. In comparison to TOPI, transpupillary illumination provides suboptimal imaging of RPE cells due to the strong signals from cone cells blocking the RPE layer. However, TOPI enhances the contrast of RPE cells due to weaker signals from cone photoreceptors due to oblique illumination. TOPI also improves the signal-to-noise ratio, enabling clearer visualization of cells. With a shorter exposure time (<10 s), TOPI provides a 4 × 4° field of view and facilitates RPE cell quantification. Therefore, TOPI can be valuable for assessing RPE in cases of IRDs primarily affecting the RPE, as well as in IRDs with photoreceptor loss where the RPE remains unaffected [58,59,60,61].

*Image acquisition:* PRs and blood vessels are imaged with a level of focus at 40–100 um and 250 um from Bruch’s membrane, respectively. Spherical equivalent and axial length correction is required for image acquisition and analysis [44]. An undilated exam provides a better picture by tackling ocular aberrations and helping in better fixation. A locus of the retina is selected, and an internal target is provided for fixation. A single focused area or montage of the fields can be acquired to assess cone mosaics.

*Image analysis:* Photoreceptor mosaics can be analyzed manually or by semi-automated/automated inbuilt software. The manual counting is time-consuming and requires a high-clarity image. In the automated software, the rtx1™ (Imagine Eyes; Orsay, Paris, France) has AODetect, with two different software for the analysis of photoreceptors and blood vessels. Reflection of the cones and their waveguiding properties affects the intensity of the photoreceptor signals and the quality of the image during analysis [62,63,64,65]. Region of interest (ROI) is chosen over the high-resolution image with fixation coordinates (x and y), and the selected area is used for analysis [44,66]. The parameters analyzed in the cone mosaic include cone density, spacing, and diameter. Voronoi cell is identified based on the highest pixel value within the PRs’ center. There are color-coded Voronoi domains based on the reflectivity of neighboring structures. Higher cone densities will have hotter colors [44,66]. Cone density refers to the number of Voronoi cells in the total area of the region of interest. Cone spacing also refers to the intercell distance measured as the distance between the center of each Voronoi cell. Other less-often-used parameters include cellular regularity, nearest/farthest neighbor distance, number of neighbors regularity, and percentage of hexagonality [44,52].

The parameters assessed in AO blood vessel imaging include wall thickness, wall-to-lumen ratio, lumen diameter, cross-sectional area of the vascular wall, architecture of the walls and changes in blood flow, bifurcation angle in the vascular branching, vessel tortuosity, capillary network, and parafoveal perfusion [44,67,68]. AO OCTA can quantitatively image choriocapillaris. Factors affecting blood vessel image acquisition include the region of interest, vessel branching, distance from the optic nerve, and motion artifacts [44].

***AO in healthy eyes:*** A large study including 2263 healthy eyes at 16–17 years of age imaged 2° temporal to fovea by rtx1™, imagine eyes. The cone density range was 14,765–39,099 cones/mm^2^ (mean 30,007) and with an increase in axial length of 1 mm; there was a decrease in cone density of 2855 cones/mm^2^ [69]. Other studies by AO-FIO have also reported a similar mean cone density of 21,214 cones/mm^2^ [70] and 31,438 cones/mm^2^ [65] at 2° temporal to the fovea. Another study involving healthy subjects (111 eyes) with a wide range of ages (6 to 78 yrs) showed a significant decline in perifoveal cone density with an increase in age and axial length with no affection by choroidal or retinal thickness. The range of cone density found was 25,409–26,924 cells/mm^2^. Automated and manual counting showed a decrease of 104 cells/mm^2^ and 116 cells/mm^2^ per year, respectively [51]. Foveal cone density by AO-SLO reported a range of 136,132 to 247,061 cones/mm^2^ (age 19–29 yrs). This highest cone density at the fovea decreased at parafoveal eccentricities (55% of peak at 150 μm eccentricity and one-third of peak at 300 μm eccentricity) [71].

### 2.3. Role of AO in IRDs (Table 2)

**Figure 3:**
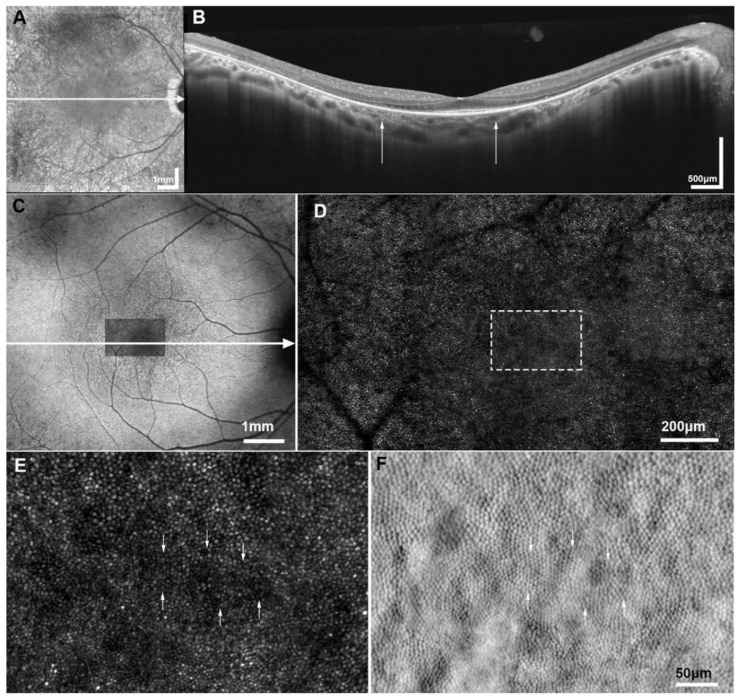
Multimodal imaging of retinitis pigmentosa: (**A**) Infrared reflectance (IR) fundus photograph of a subject with X-linked retinitis pigmentosa associated with *RPGR* gene. The white arrow represents the section of the optical coherence tomography (OCT) presented in (**B**). (**B**) Horizontal transfoveal OCT line scan, with the white arrows indicating the width of the corresponding AOSLO imaged area in (**D**). (**C**) Fundus autofluorescence imaging, with the confocal AOSLO (cAOSLO) imaged area (**D**) superimposed over the foveal avascular zone, and the white arrow represents the section presented in the OCT scan (**B**). (**D**) cAOSLO imaging reveals a disrupted waveguiding mosaic, not as uniform in appearance as in a healthy subject. (**E**) Magnification of cAOSLO over the estimated foveal center (marked with a white dashed square in (**D**)) shows irregularly waveguiding cones, which appear dim (some are indicated with white arrows); and (**F**) the corresponding split detection AOSLO in exact spatial registration showing relatively healthy-appearing cone inner segments; the white arrows indicate the corresponding inner segments for the irregularly waveguiding cones identified with white arrows in (**E**). {Reprinted under Creative Commons CC BY 4.0 license [42] (Georgiou, M., et al., Adaptive optics imaging of inherited retinal diseases. *British Journal of Ophthalmology*, **2018**, *102*, 1028–1035.)}.

*Rod cone dystrophies (Figure 3):* The central cone density is a crucial parameter to detect early changes and monitor progression. Significant changes in the cone mosaic have been noted in the presence of good vision, and a normal FAF and OCT (Table 2) [39,44,72,73,74]. Cone spacing has been negatively correlated with visual acuity. The thinning of ONL and loss of PRs in OCT have been correlated with reduced cone density and blurred cones. The outside edge of the hyperautofluorescent ring in FAF is noted with a total loss of cones. Split detector AOSLO has shown an intact inner segment of PRs at the transition zone of the hyperautofluorescent ring in FAF, wherein AO-CSLO showed missing cones [75]. Visual acuity, foveal sensitivity, and PR (inner and outer segment) thickness are inversely related to cone spacing. Alternatively, higher cone densities are related to good vision and foveal sensitivity [72,76]. An increase in cone spacing and reduced cone density by ~40 to 60% of the average normal is reported to be the threshold beyond which visual sensitivity and visual acuity are reduced [72,74,75,76].

The cone parameters are noted to be a good marker of disease progression when assessed 6 monthly in a stable-looking FAF and OCT. The cone density measure can show the progression even before the onset of changes in retinal sensitivity by microperimetry [77,78]. Gene-specific monitoring of RP like *ABCA4*, *RPE65*, *CNGA1*, *RDH12*, *RPGR*, *CRB1*, *NR3E3*, *POC1B*, *CLRN1*, *EYS*, and *RP1L1* [39,44,79], etc., have clearly shown that AO can detect the changes much earlier than currently available structural imaging (FAF, OCT) and functional tests (visual acuity, contrast vision, microperimetry, multifocal ERG). Common syndromic genes like *USH2A*, *MYO7A*, and *BBS*, which cause RP, are also studied. There is disputing evidence on cone parameters between syndromic and non-syndromic RP with a few studies showing more affected cone parameters in syndromic RP [39,44].

Clinical trials have used the cone density parameter to assess the efficacy of results. A case report evaluated a 15-year-old patient post-Luxturna gene replacement therapy. At week 5 to 3 months post-therapy, cone mosaic was noted to be better differentiated at the site of injection, superior to fovea and fovea, whereas the adjacent and untreated areas remained same in cone mosaic appearance [41]. However, the absolute cone density values were not highlighted in the study.

**Figure 4:**
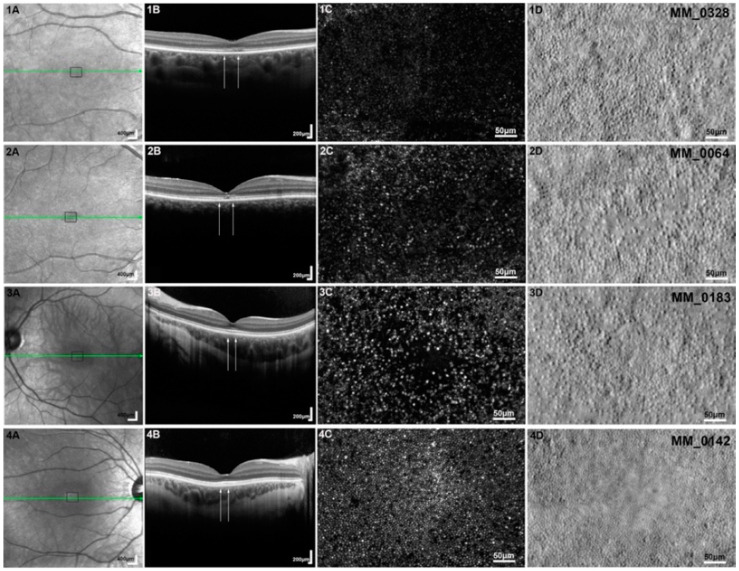
Adaptive optics scanning laser ophthalmoscopy (AOSLO) imaging of the cone dysfunction syndromes: Column (**A**) shows the infrared reflectance (IR) fundus photographs for each subject (**1**–**4**). The green arrow represents the section in which the optical coherence tomography (OCT) (Spectralis HRA + OCT, Heidelberg Engineering, Heidelberg, Germany) presented in column (**B**) is taken; the black square represents the 450 μm × 300 μm region of interest imaged with AOSLO, which is presented in columns (**C**,**D**). Column (**B**) shows OCT horizontal scans through the fovea and the white arrows mark the corresponding AOSLO area (450 μm wide). Column (**C**) depicts confocal AOSLO (cAOSLO) and column (**D**) split detection (SD) AOSLO. Subjects (**1**) and (**2**) have achromatopsia associated with *CNGB3* and *CNGA3* gene mutations, respectively. (**1C**,**2C**) Dark spaces are observed, due to the loss of cone waveguiding properties, which correspond to visible foveal cone inner segments in (**1D**,**2D**), respectively, with a substantial difference in cone numerosity between the two subjects. (**3**) A molecularly confirmed subject with blue cone monochromacy. (**3C**) Dark foveal center, with a sparse array of large bright spots, which are believed to be S cones, immediately surrounding it. (**3D**) Remnant inner segment structure. (**4**) A molecularly confirmed subject with Bornholm eye disease (LIAVA haplotype). (**4C**) All cones are resolved in cAOSLO, with a few apparent non-waveguiding cones (dark spaces). (**4D**) SD-AOSLO does not resolve foveal inner segments due to the better-preserved mosaic (smaller cone diameters and tighter packing geometry) compared with the other cone dysfunction syndromes. {Reprinted under Creative Commons CC BY 4.0 license [42] (Georgiou, M., et al., Adaptive optics imaging of inherited retinal diseases. *British Journal of Ophthalmology*, **2018**, *102*, 1028–1035.)}.

*Cone dysfunction disorders (Figure 4):* There are specific features (Table 2) noted in AO for achromatopsia (ACH) and blue cone monochromacy [80,81,82,83,84]. Dark cones found in ACH on AOSLO are noted to have residual inner segments in split detector AO. The correlation of EZ and ONL to cone density in ACH is different than what is seen in RP. *CNGA3* ACH study did not show any relation in severity of EZ disruptions with cone density. Whilst in *CNGB3 ACH*, higher peak cone density was related to better EZ preservation in OCT. ONL thickness was not correlated to peak cone density unlike RP [80,85]. *GNAT2* ACH had better cone preservation than the most common causative genes for ACH (*CNGA3* and *CNGB3*) [86]. A follow-up study of 2–3 years has shown no changes in peak foveal density in CNGB3 ACH [81,87]. Blue cone monochromacy can have abnormal S cone development and packaging at the fovea and parafoveally [88]. Bradyopsia due to *RGS9/R9AP* and oligocone trichromacy have similar clinical and standard ERG findings; however, AOSLO showed a maintained cone mosaic in bradyopsia and reduced cone mosaic in oligocone trichromacy at the fovea [89]. Nystagmus and inability to fixate are the major limiting factors in cone dysfunction syndromes, which precludes from obtaining high-quality images.

*Macular dystrophies (Table 2):* Stargardt (*ABCA4*) and Stargardt-like macular dystrophies are described to have predominant AO changes in parafoveal regions. AO parameters like cone density had better agreement with microperimetry than SD-OCT structural parameters [90,91,92,93]. Hyper-AF areas in Stargardt dystrophy are noted to have a starry night cone mosaic appearance with increased spacing. Normal FAF regions are noted to have increased cone spacing [44,75].

Best vitelliform macular dystrophy and autosomal recessive bestrophinopathy due to *BEST1* mutations have been studied for AO changes in a few reports. Cone mosaic disruption was noted in the areas of active retinal lesions, whereas the surrounding normal areas showed preserved mosaic with normal cone density [94,95,96]. Interestingly *IMPG2* and *PRPH2* variants causing vitelliform dystrophies had higher cone density reduction and higher RPE loss, respectively, when compared to *BEST1*-associated bestrophinopathies [97]. Specific features (Table 2) in occult macular dystrophy due to *RP1L1* [98,99,100] and Bietti crystalline dystrophy are reported [60,101,102].

Miscellaneous IRDs: Eyes with fundus albipunctatus due to RDH5 variants had an irregular and disrupted cone mosaic, lower cone density, lower reflectance, and increased cone spacing. [103,104]. In X-linked retinoschisis (XLRS), outer plexiform layer abnormalities were noted in a normal-looking region. Other AO features in XLRS include reduced cone density and increased cone diameter.

AO in choroideremia (CHM) has shown changes in the cone mosaic at the transition zone (Table 2) [105,106]. Other features of CHM in AO include ill-defined cone edges, groups of cones with high reflectance bubble-like hyperreflective spots with dark edges, large remnants of cone inner segments within outer retinal tubulations, widespread large RPEs, choriocapillaris flow voids in areas of increased cone spacing, and disruption of the RPE blood barrier in AOICG [105,107].

***Challenges and Future Directions:*** AO has several challenges based on the type of system used. AOSLO has better resolution and can image central fovea. However, AOSLO is not practical for a day-to-day clinic due to its time-consuming nature with multiple scan acquisition, the need for a montage protocol, and high cost. AOFIO is more feasible practically but cannot image the central 2° cones and also has less transverse resolution. The quality of the cone mosaic is affected by inherent properties of the cones like cone reflectivity and their waveguiding properties. It is also affected by other ocular factors like eye motions, very low vision, and malfixations and the presence of nystagmus, corneal aberrations, and cataract and central macular pathologies like cystoid changes, epiretinal membrane, and scarring [39,42,43,44]. Another important consideration for analyzing progression is difficulty in imaging the same area in follow-up scans with a specific ROI as in baseline. The ROI is easily affected by minimal eye movements, shifting the center in the AO montage leading to erroneous cone mosaic measurements. A low-quality image can mislead to a disease-related change. In fact, various IRD studies by different groups have shown that about 50–80% of the patients were excluded due to one or more of the above-mentioned challenges. Imaging of rod PRs is difficult as rods are less waveguiding than cones and there is a limited field of AO imaging overall [108,109].

In summary, an IRD patient with a spared central vision without nystagmus and clear optical media would be an ideal candidate for AO. The challenges of imaging the exact area can be taken care of by blood vessel-guided reference points, aligning the IR/FAF/SLO over AO montage images, eye tracking, correcting eye drifts with real-time stabilization, enface scan overlay, and AO OCT-guided foveal alignment. Over the years, when imaged properly AO has emerged as the best structural assessment tool. Various modifications have helped in addressing the challenges and allowing a structural endpoint for clinical trials in the early stages of IRD. AO-OCT and AO-microperimetry overlay to assess point-to-point structure–function correlation would be useful. Standardization of image acquisition and analysis protocol is necessary.

### 2.4. Near-Infrared Auto-Fluorescence (NIR-AF) and Near-Infrared (NIR)Reflectance

NIR-AF is based on melanin and related pigments located within the RPE and the choroid. Melanin in the RPE exhibits fluorescence properties by absorbing an NIR wavelength of 787 nm and emitting at 830 nm NIR wavelength. Red wavelengths are longer than the other wavelengths in the visible spectrum, scatter less, and are minimally absorbed by other pigments such as blood and melanin. As red wavelengths are minimally absorbed, a higher amount of reflected light is available for imaging, and it is efficient for visualizing structures deeper in the RPE. Due to the minimal absorption of the NIR-AF signal by macular pigments, greater visualization of foveal details is possible compared to short-wavelength autofluorescence (SW-AF) [110,111]. The wavelength used in NIR reflectance imaging is between 804 and 895 nm. A substance will respond to infrared illumination based on its ability to absorb, reflect, and/or disperse light. The lens, melanin, water, macular pigment, hemoglobin, and oxygenated hemoglobin are the primary absorbers found in the eye. All absorbers except macular pigment have reduced absorbance when exposed to infrared wavelengths; hence, the reflectance from the ocular fundus is substantially higher in the near-infrared than in shorter wavelengths [111,112].

***Technique and image acquisition:*** NIR-AF with confocal SLO (Heidelberg Engineering, HRA 2) uses low illumination light, leading to improved patient cooperation during image acquisition, especially in young children and photophobic patients. On the other hand, SW-AF uses bright light leading to patient discomfort, squinting, and tearing of eyes, even more so in photophobic patients, thereby reducing image quality. Owing to better contrast, affected and normal retinal/choroidal tissues can be better distinguished on NIR-AF images [111,113]. Difficulty in NIR-AF interpretation arises in cases of RPE atrophy as the unmasking of underlying choroidal signal leads to hyperautoflourescence due to the ‘window’ effect [114].

***NIR-AF in healthy eyes****:* NIR-AF shows fovea as hyperautofluorescent due to higher RPE melanin. Contrary to this, the fovea is often seen as a hypoautofluorescent area on SW-AF because of blue light absorption by macular pigments. Normally, the optic disc and blood vessels appear dark in NIR-AF and choroidal blood vessels can be visualized as dark structures [110]. NIR-AF enables qualitative imaging of the melanin by two-dimensional en face images.

***NIR-AF in IRDs:*** In retinitis pigmentosa (RP), a central area of preserved autofluorescence (APA) is often seen. The hyperautofluorescent border of the APA usually corresponds to the EZ disruption and outer retinal layer thinning on SD-OCT. NIR-AF measurements are highly reliable due to the good contrast between the APA and the damaged tissue of the retina, unlike SW-AF. Thus, NIR-AF is preferred to monitor disease progression and precedes the formation of fluorophores that lead to the SW-AF signal [115,116].

In Stargardt disease (Table 2), the NIR-AF signal is low within the area of central EZ loss. Flecks on NIR-AF appear hypoautofluorescent while on SW-AF, it appears hyper or iso-autofluorescent. Areas of low signals in SW-AF are usually smaller than areas of low NIR-AF signals. Hence, lesions are more clearly defined in NIR-AF pictures than in SW-AF. Areas of reduced NIR-AF signal correspond with EZ and RPE loss on SD-OCT. EZ loss is typically seen only when NIR-AF is either absent or very low, suggesting RPE cell atrophy happening prior to photoreceptor cell degeneration [117].

In choroideremia (CHM), lipofuscin-related autofluorescence is retained longer than melanin-related AF. Residual islands of normal retina appear much larger on SW-AF than NIR-AF imaging. NIR-AF signal loss at the edge of retinal degeneration denotes impending RPE and photoreceptor loss. NIR-AF signal attenuation is seen in the early stages of RPE alteration in CHM when the SWAF signal, photoreceptors, ellipsoid zone, and retinal sensitivity are still intact [118]. Recently studies have shown the usefulness of combining NIR-FLIO with AOSLO to understand the lifetime of fluorescence from RPE mosaic [119]. NIR-AF AOSLO with transscleral flood imaging has also helped in assessing fluorophore distribution and its relation to RPE mosaic [120].

**Near-infrared (NIR) reflectance in IRDs (Table 2):** In RP, a central hyperreflective ring is seen similar to NIR-AF. Bone spicules are seen as nonreflective areas on NIR imaging. As NIR imaging reveals the extent of the damaged retina, it can be used in conjunction with other imaging modalities to identify RP progression. Carriers of X-linked recessive RP can be screened with NIR by visualizing radial hyperreflectivity around the macula seen with a tapetal-like reflex [111,121]. In Leber congenital amaurosis (LCA), foveal hyperreflectivity is seen as an early finding before clinically visible foveal atrophy develops. Other features of LCA on NIR are foveal hypo reflectivity with adjacent alternating hyper- and hyporeflective zones and diffuse hyperreflectivity over the posterior pole. The clinical significance of these findings is unclear [122,123]. Discrete hyperreflective dots are seen in patients with fundus albipunctatus on NIR imaging referred to as target signs. These dots correspond to the yellow flecks seen clinically and hyperreflective dots seen on OCT at the level of the outer retina [124].

In Bietti crystalline dystrophy (BCD), as chorioretinal atrophy advances, retinal crystals become less apparent with the color fundus picture. Even in the presence of severe chorioretinal atrophy, NIR imaging is more specific and sensitive in identifying these crystalline deposits. Retinal crystalline material appears hyperreflective on NIR, which is often confluent and corresponds to hyperreflective material at the RPE level on SD-OCT. This unique property of NIR helps to identify BCD and differentiate it from other retinal dystrophies that can also cause crystalline-like deposits even in advanced degeneration. Sclerotic choroidal vessels in advanced BCD appear as large hyperreflective choroidal vessels on NIR imaging [112,125].

In Stargardt disease, NIR can detect flecks and outer retinal atrophy accurately. On NIR, resorbing flecks show hyporeflectivity, whereas new flecks show hyperreflectivity. On near-infrared imaging, bull’s eye maculopathy manifests as a hyporeflective ring surrounding the fovea [126,127]. In OMD, clinically undetectable changes are seen as low reflective alterations at the central macula [128].

***Future directions:*** NIR-AF and reflectance are easily available for a day-to-day clinic practice. NIR is a more comfortable imaging procedure in photophobic and pediatric IRD patients. Quantitative analysis of parameters like hyperautofluorescent ring and area of preserved autofluorescence would provide precise information to monitor IRDs. Some of the challenges include the following: a) To understand whether an increased level of RPE lipofuscin can modulate the melanin-related NIR-AF signal in a degenerative retina of IRD. b) Can NIR-AF completely replace SW-FAF or is it just a complementary tool to SW-AF? c) The contribution of choroidal melanin to NIR–AF in an advanced stage of RP with complete loss of RPE melanin needs to be explored.

### 2.5. Polarization Sensitive OCT (PS-OCT)

PS-OCT measures the polarization property of melanin (i.e., depolarization) as a functional extension of OCT. It is based on the fundamental principle that various tissues can change the polarization state of the incident light [129,130]. The light-tissue interaction or polarization can either be birefringence, diattenuation, or depolarization. Tissues that can cause birefringence include extraocular muscles, corneal stroma, retinal nerve fiber layer, Henle fiber layer, sclera, and scar tissue [129]. Depolarization can be seen in tissues that contain melanin, like iris pigment epithelium, RPE, melanotic tumors, pigment-laden macrophages, and to some extent, the choroid [129,131]. Diattenuation is negligible in biological tissues. PS-OCT provides distribution of RPE melanin depth-wise and is selective to layers of retina/choroid (3D analysis).

***Technique and image acquisition:*** Traditional PS-OCT systems use the time-domain setup, the newer ones use swept source and spectral domain set-ups [132]. The setup of PS-OCT devices is quite similar to that of conventional OCT devices. The difference being that the incident light is first passed through a polarizer that converts the light into a linearly (vertical) polarized light before entering the Michelson interferometer [129]. After being split by a beam splitter, the polarized light passes through a quarter-wave plate (QWP) oriented at 45 degrees in the sample arm, and 22.5 degrees in the reference arm (as the beam enters the QWP twice in the reference arm). This converts the linearly polarized light into a circularly polarized light. After recombination of the light from the sample and the reference arm, the 2 linear polarized states (horizontal and vertical) are separated by a polarizing beam splitter before detection. The detectors then analyze the amplitude and the phase of the final signal [129]. Since its initial description in 1992, PS-OCT technology has undergone several modifications that have increased the scan speeds, and various computational methods have been introduced that have reduced artifacts significantly [132].

**Figure 5:**
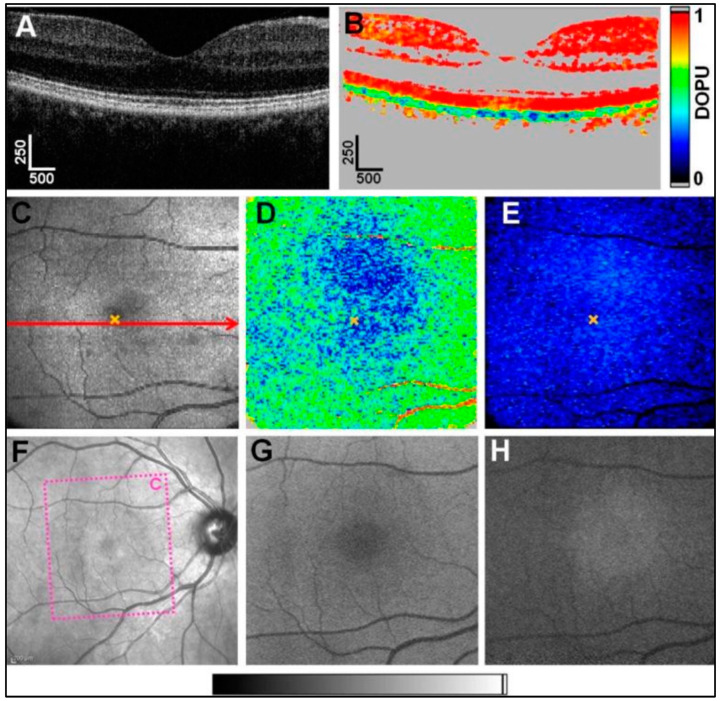
PS-OCT imaging and retinal pigmentation: (**A**) Reflectivity B-scan image showing the retinal layers. (**B**) Corresponding degree of polarization uniformity (DOPU) B-scan image. DOPU values close to 1 can be observed in most retinal layers. Lower DOPU values indicate depolarization and can be observed in the pigmented RPE and choroid. See Section 2.3 for a detailed description of DOPU calculations. (**C**) OCT fundus projection image of a volumetric PS-OCT data set of the macula. The red arrow indicates the location of the B-scan image shown in (**A**). The orange “×” marks the fovea in (**C**–**E**). (**D**) DOPU_min_ map showing the minimal DOPU value for every A-scan. The color scale encodes DOPU_min_ values ranging from 0 (blue) to 1 (red). The most severe depolarization can be observed close to the fovea. (**E**) Thickness map of segmented depolarizing pixels with DOPU < 0.7. The color scale ranges from 0 to 39 pixels per A-line. Pixel height: 3.22 µm in air. (**F**) Wide-field infrared scanning laser ophthalmoscope image. The dotted box(c) indicates the location of the PS-OCT scan. (**G**) Corresponding fundus autofluorescence image at 488 nm showing the retinal lipofuscin distribution having a minimum at the fovea. (**H**) Near-infrared autofluorescence image at 787 nm showing the retinal melanin distribution peaking at the fovea. Grey scale bar for the scanning laser ophthalmoscope image and the autofluorescence images is shown below (**F**–**H**). Scale bar dimensions in micrometers. {Reprinted with permission from [131] © Optica Publishing Group (Baumann, B., et al., Polarization sensitive optical coherence tomography of melanin provides intrinsic contrast based on depolarization. *Biomedical Optics Express*, **2012**, *3*, 1670–1683.)}.

***PS-OCT in healthy eyes:*** There are two ways of melanin quantification: (1) Degree of polarization uniformity (DOPU—Figure 5). Polarimetric entropy to assess the amount of depolarization. DOPU is sensitive to the incident state of polarization and does not have a noise-bias correction. DOPU values are close to 1 in most retinal layers under a healthy condition. Lower DOPU values indicate depolarization (seen in RPE and choroid). Polarimetric entropy is linearly proportional to melanin concentration. Entropy values for RPE are higher in the center of the macula and lower outside. The entropy of RPE melanin is noted to increase with age (increased melanolipofuscin granules) and is negatively correlated with axial length. On the other hand, the entropy of choroidal melanin is non-uniform [133]. In terms of retinal imaging, PS-OCT is particularly helpful in detecting structural alterations in the RPE (which can have similar reflectivity to other adjacent structures like the photoceptor layer) and differentiating them from scar tissues.

***PS-OCT in IRDs:*** In Stargardt disease, the RPE segmentation B-scans, DOPU images, RPE elevation maps, and depolarizing material thickness maps of PS-OCT have shown benefits in detecting morphological alterations in the outer retina and RPE (margins of RPE atrophy and RPE irregularities) [134]. Defects in the outer retina without RPE loss can also be detected using intensity-based images. RPE irregularities seen on PS-OCT correspond to flecks seen on FAF [134]. In vitelliform dystrophies, PS-OCT shows a central depolarizing signal on DOPU images with surrounding atrophy, corresponding to the subretinal accumulations [135]. Sakai et al., in their study of PS-OCT findings in eyes with retinitis pigmentosa (Table 2), used “polarimetric entropy” (rather than DOPU) to analyze the RPE, and found its value to be higher at the foveal region compared to other areas [136]. Also, the entropy values had a strong positive correlation with NIR-AF findings, while a weak negative correlation with SW-AF findings. They also noted that while the AF signals gradually reduce toward the periphery, beyond the hyperautofluorescent ring, the PS-OCT entropy showed a moderate decrease. Although PS-OCT and NIR-AF are sensitive to the degree of melanin in a tissue, RPE is not the only structure responsible for the AF signal. AF is also contributed to some extent by the photoreceptors and the choroid. PS-OCT has an added advantage over NIR-AF in this aspect that it gives more precise information about the RPE [136,137]. This could also hold an importance in future therapies involving RPE transplant. En face PS-OCT showed high-entropy areas corresponding to the densely pigmented regions of the hiPSC-RPE sheet in a study [138].

There is a newer type of PS-OCT called polarization diversity OCT (PD-OCT), which images tissue-specific melanin in retinal and choroidal areas with a wider field of view (55 to 120°). In RP, studies have shown melanin-specific information from peripheral retina and melanin migration as part of disease progression [139,140].

***Challenges and Future directions:*** Layer-specific and quantitative melanin evaluation by PS-OCT can detect RPE-melanin changes more precisely. PS-OCT melanin distribution requires age-, gender-, and axial-length-matched normative data before clinical implication in a particular IRD. How the PS-OCT melanin content correlates to the functional changes in terms of retinal sensitivity needs to be explored.

### 2.6. Optoretinography (ORG)

Optoretinography represents a groundbreaking advancement in retinal imaging, offering a non-invasive, objective method for assessing the function of the retina. Analogous to the well-established electroretinography (ERG), which measures the electrical responses of retinal cells to visual stimuli, ORG utilizes intrinsic optical signal (IOS) imaging to detect changes in properties of light within the retina that correspond to functional activity [141]. ORG is combined with OCT as an interferometric imaging technique that captures high-resolution cross-sectional images of retinal layers. It works by analyzing interference patterns generated by back-scattered light from the retina, using Fourier transform methods to extract both intensity and phase information [142,143]. While traditional OCT images are created using only the signal amplitude, the phase information, often disregarded, contains critical data about the position and movement of retinal structures. This phase-sensitive approach allows ORG to detect minuscule tissue movements, providing unparalleled sensitivity to physiological changes within photoreceptors [141,144].

***Principle:*** ORG relies on the interaction between light and the retina, specifically targeting the optical responses generated by retinal cells upon light stimulation. Initially, controlled light stimuli of specific wavelengths, intensities, and durations are directed onto the retina, aiming at activating different types of photoreceptors (rods and cones) and other retinal cells. As light penetrates the retina, it triggers biochemical and biophysical changes within the photoreceptors. These alterations in cellular activity subsequently modify the optical properties of the photoreceptors, including their refractive index and light scattering characteristics. ORG detects these intrinsic optical signals [145]. Utilizing advanced imaging techniques such as interferometry or other high-resolution methods, ORG measures minute variations in the optical path length within the retina. These variations serve as sensitive indicators of the functional responses of retinal cells to light stimuli, providing valuable insights into retinal physiology and function with high sensitivity and spatial resolution [141,144,146,147,148].

***Technique and image acquisition:*** It involves a precise sequence of steps aimed at capturing and interpreting intrinsic optical signals (IOS). Initially, controlled light stimuli are delivered through methods such as light-emitting diodes. An interferometric setup or high-resolution imaging device then detects the reflected or transmitted light from the retina, capturing phase and amplitude changes in the optical signals. These signals, indicative of photoreceptor activity, undergo detailed data acquisition and analysis. Signal processing techniques discern different IOS waveforms, particularly biphasic patterns corresponding to photoreceptor activation and recovery phases. Spatial mapping of these functional responses across the retina, facilitated by correlating IOS with specific retinal layers, elucidates the regional integrity and health of retinal function. ORG’s capability to detect subtle optical path length changes within the photoreceptor outer segment, leveraging its waveguide properties and sensitive phase-resolved OCT technology, underscores its utility in studying rapid physiological responses crucial for visual processing and monitoring retinal health [146,149]. ORG has been combined with PS-OCT and AO to explore phase-sensitive and localizing information from the retinal PRs [150]. ORGs can be phase-based (pORGs) or intensity-based (iORGs). pORGs are generated by changes in phase determined by OCT signal and changes in optical path length. Whereas changes in amplitude of backscattered light from PRs in AO-FIO represent iORGs [151,152,153].

***ORG in healthy eyes:*** In a normal eye, rapid alterations in the PR’s optical path length, are captured as biphasic waveforms, with an initial phase of rapid hyperpolarization followed by a slower recovery phase, indicative of the phototransduction process. ORG further distinguishes between optical responses originating from the outer and inner retinal layers, highlighting the distinct functional activities of these regions. This layer-specific analysis not only underscores the normal functionality of the retina but also serves as a baseline for comparison in detecting deviations associated with retinal diseases or pathologies. ORG responses increase with increasing stimulus irradiance and a steep response in amplitude is characteristic of normal cones [149].

**Figure 6:**
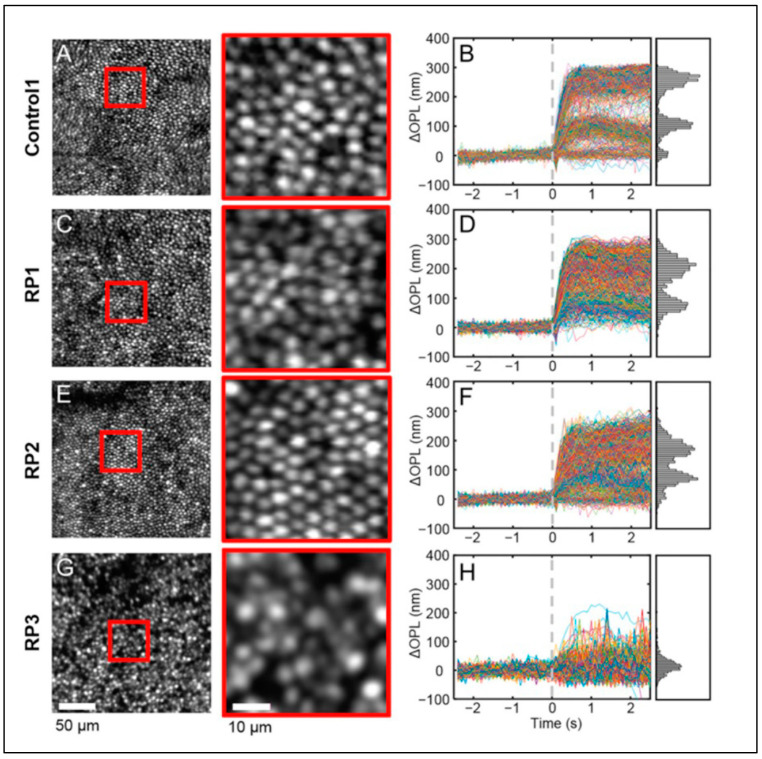
Cone photoreceptor structure and function. AO-OCT en face images of the cone mosaic at 2° eccentricity in one of the controls (**A**,**B**) and the three RP subjects: RP1 (**C**,**D**), RP2 (**E**,**F**), and RP3 (**G**,**H**). Magnified views are shown for cones in red boxes. Cone densities are 31,491 (Control1), 22,750 (RP1), 25,258 (RP2), and 14,041 (RP3) cones/mm^2^. Traces of individual cones’ ΔOPL responses to 637 nm stimulus are shown in (**B**,**D**,**F**,**H**). Traces are randomly color-coded, and histograms of peak responses (average ΔOPL from 0.75 to 1.25 s after stimulus) are shown to the right of each plot. {Reprinted under Creative Commons CC BY 4.0 license [54] (Lassoued, A., et al., Cone photoreceptor dysfunction in retinitis pigmentosa revealed by optoretinography. *Proceedings of the National Academy of Sciences*, **2021**, *118*, e2107444118.)}.

***ORG in IRDs:*** A study combined PS-OCT with AO and cone ORG to assess the cone PRs in RP (Table 2). The same study also noted the presence of isolated normal-functioning cones in an area with advanced degeneration (Figure 6) [54]. In this way, ORG can evaluate residual retinal function crucial for monitoring gene therapy outcomes in RP. Another study evaluated iORGs in RP patients and found reduced iORG amplitudes in various retinal eccentricities till 8° when compared to control. iORG amplitudes were more sensitive when compared to retinal sensitivity measured by macular integrity assessment microperimeter. Hence, ORG can be a great tool to evaluate residual retinal function and therapy outcomes in RP.

AO-ORG in patients with CHM showed a reduced and slow rise in parafoveal amplitudes compared to normal. ORG amplitudes were noted to correlate with EZ length (Table 2). A reduced ORG amplitude was also noted in cases of CHM with normal central vision and normal cone density. Higher cone densities with contiguous cone mosaic and reduced cone spacing in AO were correlated to higher ORG amplitudes [154]. As ORG technology advances, including improvements in instrumentation and data processing, its role in IRD management promises to grow, guiding personalized therapies aimed at preserving retinal function and vision [144].

***Challenges and Future Directions:*** ORG faces several critical challenges as it strives to become a mainstream tool in ophthalmic diagnostics. Standardization of techniques across different imaging systems remains a primary hurdle, requiring uniform protocols for light stimulation, data acquisition, and analysis to ensure consistent results. Integration with established clinical practices such as perimetry and ERG is crucial for validating ORG’s clinical utility and comparative efficacy. Improving spatial resolution to achieve single-cell sensitivity would enhance its ability to map retinal function with precision [147]. Furthermore, advancing image processing algorithms and anatomical modeling is essential for accurately interpreting ORG data in relation to specific retinal structures. Validation through large-scale clinical trials and technological advancements in OCT systems is needed to optimize ORG performance, ensuring robustness in early disease detection and treatment evaluation.

### 2.7. Mitochondrial Flavoprotein Fluorescence (FPF) Imaging

Mitochondrial flavoprotein fluorescence (FPF) imaging is emerging as a promising technique that measures the metabolic change as an indicator of oxidative stress in a tissue [155,156]. FPF that detects early metabolic changes can be superior to all the available functional tests.

***Principle and technique:*** Mitochondrial flavoproteins, under oxidizing conditions, emit green (520–540 nm) AF when excited by blue (430–470 nm) light. Emitted green signals are quantified to assess oxidative stress. FPF signals are quantified as either intensity or heterogeneity. Global signal strength represents intensity and variation in the intensity denotes heterogeneity [156,157,158].

Currently, FPF imaging (OcuMet Beacon third-generation device) is under investigational use only. Studies have evaluated FPF intensities and heterogeneities in normal healthy eyes and in retinal diseases (DR, AMD, CSCR). Higher FPF heterogeneity is noted in various retinal diseases associated with oxidative stress like DR, AMD, and CSCR and neurodegenerative conditions [155,156,159,160].

***FPF Imaging in IRDs*:** Emerging studies highlight that FPF may be an early indicator of oxidative stress in various IRDs. Its role in RP is described as early as 2008 by Elner et al., who showed a single patient of RP with increased flavoprotein autofluorescence when compared to healthy control [160]. Recently in a large cohort of patients with genetically confirmed IRDs, FPF intensity and heterogeneity were significantly increased in patients with rod–cone dystrophy, Stargardt disease, and Bardet–Biedl syndrome and in Mitochondrial ATP synthase mutation disorder compared to age-matched controls. These data strengthen the concept of oxidative stress in IRD patients. FPF heterogeneity was also found to correlate with FAF lesions, suggesting its relevance as a clinically significant imaging marker. However, there was no significant correlation between visual acuity and FPF imaging parameters in the study [158].

**Advantages and Challenges of FPF:** FPF offers several benefits, such as noninvasively providing objective, functional information on early alterations in mitochondrial metabolism. Exciting future avenues include its potential use with therapeutics targeting oxidative stress and its integration with artificial intelligence-based analyses of ocular biofluids to detect oxidative stress and OCT imaging to offer personalized patient care [161,162]. Currently, the device is not widely available. There are no universal standards for image processing parameters and healthy FPF reference values. Additionally, factors such as age, gender, cataract presence, corneal pathology, and ocular or systemic comorbidities can increase variability in FPF signals decreasing reliability [155].

### 2.8. Retinal Oximetry

Retinal vessel oximetry provides information on the metabolic health of the retina by assessing oxygen saturation (SO_2_) in the retinal vessels. IRDs are known to have an increase in SO_2_ and less oxygen demand. The SO_2_ parameters are assessed for both arteries (A-SO_2_) and venules (V-SO_2_) along with the caliber of the vessels. SO_2_ is noted to be correlated with structural and functional parameters [163,164,165,166].

The imaging can be performed by oximetry tool of retinal vessel analyzer (RVA; Imedos UG, Jena, Germany) with a connection to a fundus camera or by a dual-wavelength non-invasive retinal oximeter Oxymap T1 (Oxymap ehf., Reykjavik, Iceland). Red and green channel illumination will be set up with imaging centered around the optic disc [167,168]. A recent study on 120 healthy eyes revealed normative data for SO_2_. The median age was 47 years, mean A-SO_2_ was 92.2 ± 6 3.7%, and V-SO_2_ was 55.6 ± 6 6.3%. With age, there was a decrease in V-SO_2_ affecting the average difference between A-SO_2_ and V-SO_2_ [164].

A study by Türksever et al. showed that patients with RCD had higher average SO_2_ for both arteries and venules, increased oxygen exposure, and narrow diameter of peripapillary vessels when compared to controls [167]. Another study compared the mean A-SO_2_ and V-SO_2_ values in RCD, which were significantly higher when compared to cone–rod dystrophies, inherited maculopathies, and controls [169]. These findings indicate that RCDs have severely affected oxygen metabolism with high oxygen demand among all IRDs [170]. A pilot study on 15 RP patients compared the difference in A-SO_2_ and V-SO_2_ to the structural parameter of central macular thickness, which was positively correlated. The mean A-SO_2_ (99.3%) and V-SO_2_ (66.8%) were significantly higher compared to controls—mean A-SO_2_ (92.4%) and V-SO_2_ (54.0%) [166].

Overall retinal oximetry looks promising in understanding the pathophysiology and detecting metabolic change and retinal SO_2_ in both early and late stages of IRD. All the studies have shown significant SO_2_ change in RCD. Hence, it can help in monitoring all types of RCD irrespective of genetic and phenotypic heterogeneity. Thus, retinal oximetry can be a very good tool for assessing progression in advanced stages and monitoring probable changes in metabolic demand post any therapeutic intervention. The retinal oximetry device is not available for clinical use currently. Its wider usage is necessary to understand its usefulness in various stages of a gene-specific IRD in a larger cohort.

### 2.9. Detection of Apoptosing Retinal Cells (DARC)

Detecting apoptosis and cell stress early offers a promising strategy for diagnosing neurodegenerative diseases before irreversible damage occurs and for monitoring treatment responses earlier than existing endpoints [171,172,173]. DARC technology uses a fluorescent-labeled annexin V protein, administered via intravenous injection, to identify apoptosis [174]. In early apoptosis and cell stress, structural changes occur in the cell membrane: phosphatidylserine (PS) moves from the inner leaflet of the cell membrane to the outer leaflet, acting as an “eat me” signal for resident phagocytes [175]. This externalization of PS is reversible, allowing stressed cells to return to a healthy state if targeted appropriately by treatment. Annexin V binds to exposed PS with high affinity. These individually labeled cells are then visualized as white spots (apoptotic cells) on CSLO retinal images captured using standard imaging equipment. DARC technology provides a method not only to monitor disease activity (DARC count) but also to assess treatment efficacy (reduction in DARC count) [176].

In normal eyes, DARC imaging typically shows a minimal number of apoptotic retinal cells, reflecting the baseline level of cellular turnover and apoptosis. Studies have shown an increased DARC count in glaucoma for retinal ganglion cells and in AMD. Geographic atrophy progression is also predicted with the assistance of DARC [176,177,178,179]. However, the role of DARC in IRDs is yet to be explored. Combining DARC with other diagnostic tools, such as PS-OCT, AO, and artificial intelligence-based analyses, could enhance its utility in personalized patient care. Discrepancies in DARC spot counts and the current invasive approach also need to be addressed.

## 3. Summary

Advances in IRD imaging play an essential role in providing diagnosis, counseling, and monitoring progression. Imaging in IRDs is advancing toward cell-specific structural and functional/metabolic assessments. These newer imaging modalities not only help in better monitoring of the disease/therapeutic effect but also provide newer concepts in the pathophysiology of IRDs. However, establishing the reference data across different ages is one of the limitations of these advanced imaging modalities. A combination of cellular imaging with functional assessments (e.g., AO, PS-OCT, FLIO, ORG) is promising and could help stage disease severity. Understanding the metabolic stress at the level of photoreceptors and RPE is far superior to structural and functional imaging to detect the earliest changes in the disease process. Currently, among the imaging modalities described in this review, NIR-AF and reflectance are the only imaging modalities used in routine clinical practice. AO-SLO, AO-FIO, AO-OCT, FLIO, and PS-OCT are the remaining investigative tools that are close to clinical use. ORG, mitochondrial imaging, retinal oximetry, and DARC require further investigation to define their clinical application and usefulness in IRDs.

## Figures and Tables

**Table 1:** Showing the characteristics of fluorescence lifetime in normal healthy eyes.

Region of the Retina	Fluorescence Lifetime	Approximate Fluorescence Lifetime Values	Color Code	The Structure Responsible for the Fluorescence Lifetime
(a) Centre of the macula (as per ETDRS * grid)	Shortest	50–200 ps * in SSC * 100–240 ps * in LSC *	Red	Central macular pigment (Xanthophyll)
(b) Outside of the fovea	Intermediate	Varies in between shortest and longer fluorescence lifetimes	Orange, yellow, green	Retinal pigment epithelium (Lipofuscin)
(c) Retinal vessels and Optic nerve head	Longer	1250 ps * in SSC * 1000 ps * in LSC *	Blue	Collagen and elastin

* ETDRS, Early-Treatment Diabetic Retinopathy Study; ps, picosecond; SSC, short spectral channel; LSC, long spectral channel (LSC).

**Table 2:** Highlight features of newer advanced imaging modalities studied for IRD.

Imaging Type	IRD Studied	Imaging Features
1. FLIO *	Stargardt disease	New flecks; Shorter fluorescence lifetimes Old flecks and atrophic areas; Longer fluorescence lifetimes
	Retinitis pigmentosa	Longer and shorter fluorescence lifetimes outside and inside of the hyperautofluorescent ring respectively
	Choroideremia	Areas with RPE * and photoreceptor loss; Longer fluorescence lifetime Areas with RPE * loss but preserved photoreceptors; Shorter fluorescence lifetime
2. AO *	Retinitis pigmentosa	Central macula: Reduced cone density, increased cone spacing, irregular mosaic, disruption of the mosaic with dark patches from perifovea to parafoveal eccentricities, and enlarged cone photoreceptors Outer to hyperautofluorescent ring; Total loss of cones
	Achromatopsia	Central macula; Abnormally large cones, reduction in peak cone density, dark cones with reduced reflectance and increased cone spacing
	Blue cone monochromacy	Residual cones are non-waveguiding with decreased total cone density
	Stargardt disease	Parafoveal cone mosaic; Enlarged cones, reduced cone density, and increased spacing Starry night cone mosaic appearance
	Occult macular dystrophy	Patchy cone mosaic, reduced cone density, enlarged cones with preserved RPE *
	Bietti crystalline dystrophy	Irregular cone spacing, reduced cone density, and enlarged RPE cell with altered fluorescence pattern
	Fundus albipunctatus	Retinal flecks; Hyperreflective mosaics with hypo reflective rings and loss of cones/RPE * at the site
	Choroideremia	Intact cone mosaic up to the border of retinal atrophy followed by a sharp transition, microcysts, hyperreflective clumps, and variable parafoveal cone density
3. NIR-AF *	Stargardt disease	Early flecks; Hypoautofluorescent Clearly defined fleck and atrophy border Areas with EZ * and RPE * loss; Reduced signal
	Choroideremia	Autofluorescence signal attenuation seen in very early stages of RPE and photoreceptor loss
4. NIR * reflectance	Retinitis pigmentosa	Hyperreflective ring and nonreflective bony spicules
	Fundus albipunctatus	Flecks; Hyperreflective dots (target signs)
	Bietti crystalline dystrophy	Crystalline deposits; Hyperreflective even in advanced stages
5. PS-OCT *	Retinitis pigmentosa	Higher polarimetric entropy of the RPE at the foveal region Gradual and moderate decrease in entropy from parafoveally to periphery
6. ORG *	Retinitis pigmentosa	Decreased cone responses and optical path length with an increase in the severity of RP Short wavelength cone ORGs will be more affected
	Choroideremia	Better ORG amplitude was positively correlated to EZ-RPE */Bruch’s membrane length, foveal sensitivity, and cone density

* FLIO; fluorescence lifetime imaging ophthalmoscopy, RPE; retinal pigment epithelium, AO; adaptative optics, NIR; near infra-red, AF; autofluorescence, EZ, ellipsoid zone; PS-OCT; polarization sensitive OCT, ORG; optoretinography.

## Data Availability

Available from the corresponding author on request.
